# Supplementary material for: Multidimensionality of tree communities structure host-parasitoid networks and their phylogenetic composition
Source: eLife. 2025 Feb 25;13:RP100202. doi: 10.7554/eLife.100202 (PMC11856933; doi:10.7554/eLife.100202)
Supplement: Supplementary file 1. [file elife-100202-supp1.docx]

**Supplementary File 1**

***Multi-dimensionality of tree communities structure host-parasitoid networks and their phylogenetic composition***

***Wang et al.***

Supplementary file 1a. Hymenopteran species composition and abundance across all study plots species used in parafit analysis were marked.

| Family | Species | Abundance | Role | Parafit |
| --- | --- | --- | --- | --- |
| Colletidae | *Hylaeus* sp.1 | 62 | Host |  |
| Megachilidae | *Heriades sauteri* | 201 | Host | √ |
| Megachilidae | *Hoplitis carinotarsa* | 382 | Host | √ |
| Megachilidae | *Megachile strupigera* | 326 | Host | √ |
| Megachilidae | *Megachile monticola* | 18 | Host | √ |
| Megachilidae | *Megachile pseudomonticola* | 10 | Host | √ |
| Megachilidae | *Megachile rixato*r | 131 | Host | √ |
| Megachilidae | *Megachile sculpturalis* | 363 | Host | √ |
| Megachilidae | *Megachile spissula* | 313 | Host | √ |
| Megachilidae | *Megachile dinura* | 7 | Host | √ |
| Megachilidae | *Osmia taurus* | 121 | Host | √ |
| Megachilidae | *Trachusa staabi* | 83 | Host |  |
| Vespidae | *Allorhynchium chinense* | 83 | Host | √ |
| Vespidae | *Anterhynchium flavomarginatum* | 8644 | Host | √ |
| Vespidae | *Discoelius nigridypeus* | 15 | Host | √ |
| Vespidae | *Discoelius* sp.1 | 4 | Host |  |
| Vespidae | *Discoelius wangi* | 41 | Host | √ |
| Vespidae | *Epsilon fujianensis* | 483 | Host | √ |
| Vespidae | *Eumenes quadratus* | 17 | Host | √ |
| Vespidae | *Eumenes sillicus* | 4 | Host |  |
| Vespidae | *Eumenes* sp.1 | 3 | Host |  |
| Vespidae | Eumeninae sp.1 | 2 | Host | √ |
| Vespidae | Eumeninae sp.2 | 19 | Host | √ |
| Vespidae | Eumeninae sp.3 | 3 | Host |  |
| Vespidae | Eumeninae sp.4 | 11 | Host |  |
| Vespidae | Eumeninae sp.5 | 1 | Host |  |
| Vespidae | Eumeninae sp.8 | 8 | Host | √ |
| Vespidae | *Euodynerus notatus* | 54 | Host | √ |
| Vespidae | *Euodynerus quadrifasciatus* | 49 | Host | √ |
| Vespidae | *Orancistrocerus aterrimus* | 34 | Host | √ |
| Vespidae | *Orancistrocerus drewseni* | 367 | Host | √ |
| Vespidae | *Pararrhynchium ornatum* | 16 | Host |  |
| Vespidae | *Pareumenes quadrispinosus* | 389 | Host | √ |
| Sphecidae | *Chalybion japonicum* | 53 | Host | √ |
| Sphecidae | *Isodontia nigella* | 109 | Host | √ |
| Sphecidae | *Sceliphron deforme* | 90 | Host | √ |
| Crabronidae | *Passaloecus insignis* | 27 | Host | √ |
| Crabronidae | *Pison atripenne* | 31 | Host |  |
| Crabronidae | Crabronidae sp.1 | 14 | Host |  |
| Crabronidae | *Trypoxylon bicolor* | 91 | Host | √ |
| Crabronidae | *Trypoxylon* sp. | 26 | Host | √ |
| Pompilidae | *Auplopus kuarensis* | 371 | Host | √ |
| Pompilidae | *Auplopus carbonarius* | 141 | Host |  |
| Pompilidae | *Auplopus* sp.1 | 1 | Host | √ |
| Pompilidae | *Auplopus* sp.2 | 29 | Host | √ |
| Pompilidae | *Auplopus* sp.3 | 13 | Host | √ |
| Pompilidae | *Auplopus* sp.4 | 66 | Host | √ |
| Pompilidae | *Auplopus* sp.5 | 1 | Host |  |
| Pompilidae | *Auplopus* sp.6 | 92 | Host | √ |
| Pompilidae | *Auplopus* sp.7 | 3 | Host | √ |
| Pompilidae | *Auplopus* sp.9 | 4 | Host |  |
| Pompilidae | *Auplopus* sp.10 | 2 | Host |  |
| Pompilidae | *Auplopus* sp.11 | 10 | Host |  |
| Pompilidae | *Auplopus* sp.12 | 5 | Host |  |
| Pompilidae | *Deuteragenia ossarium* | 110 | Host | √ |
| Pompilidae | Pompilidae sp.1 | 3 | Host |  |
| Braconidae | Braconidae sp.1 | 32 | parasitoid | √ |
| Eulophidae | *Chaenotetrastichus semiflavus* | 4 | parasitoid |  |
| Eulophidae | Eulophidae sp.1 | 1 | parasitoid |  |
| Eulophidae | *Kocourekia* sp.1 | 11 | parasitoid |  |
| Eulophidae | *Melittobia australica* | 1 | parasitoid |  |
| Eulophidae | *Melittobia clavicornis* | 42 | parasitoid | √ |
| Eulophidae | *Melittobia sosui* | 444 | parasitoid | √ |
| Eulophidae | *Melittobia* sp.1 | 1 | parasitoid |  |
| Eulophidae | *Melittobia* sp.2 | 2 | parasitoid | √ |
| Chrysididae | Chrysidinae sp.1 | 76 | parasitoid | √ |
| Chrysididae | Chrysidinae sp.2 | 4 | parasitoid |  |
| Chrysididae | Chrysidinae sp.3 | 2 | parasitoid |  |
| Chrysididae | Chrysidinae sp.4 | 2 | parasitoid | √ |
| Chrysididae | Chrysidinae sp.5, | 6 | parasitoid | √ |
| Chrysididae | *Chrysis principalis* | 1244 | parasitoid | √ |
| Chrysididae | *Chrysura* sp.1 | 9 | parasitoid | √ |
| Encyrtidae | *Copidosoma* sp.1 | 1 | parasitoid | √ |
| Ichneumonidae | *Dusona* sp.1 | 35 | parasitoid | √ |
| Ichneumonidae | Ichneumonidae sp.1 | 22 | parasitoid | √ |
| Ichneumonidae | Ichneumonidae sp.5 | 4 | parasitoid |  |
| Ichneumonidae | Ichneumonidae sp.6 | 2 | parasitoid |  |
| Ichneumonidae | Ichneumonidae sp.7 | 1 | parasitoid |  |
| Ichneumonidae | *Picardiella melanoleuca* | 90 | parasitoid | √ |
| Leucospidae | *Leucospis japonicas* | 27 | parasitoid | √ |
| Leucospidae | *Leucospis* sp.1 | 6 | parasitoid | √ |
| Trigonalyidae | *Lycogaster flavonigrata* | 4 | parasitoid | √ |
| Trigonalyidae | *Lycogaster violaceipennis* | 597 | parasitoid | √ |
| Mutillidae | Mutillidae sp.1 | 8 | parasitoid | √ |
| Mutillidae | Mutillidae sp.2 | 1 | parasitoid |  |
| Perilampidae | Perilampidae sp.1 | 3 | parasitoid | √ |
| Torymidae | Torymidae sp.1 | 28 | parasitoid | √ |
| Megachilidae | *Coelioxys crassiventris* | 75 | parasitoid | √ |
| Megachilidae | *Coelioxys fenestrate* | 52 | parasitoid | √ |
| Megachilidae | *Coelioxys* sp.1 | 1 | parasitoid | √ |
| Megachilidae | *Euaspis basalis* | 32 | parasitoid | √ |
| Megachilidae | *Euaspis Polynesia* | 4 | parasitoid |  |
| Gasteruptiidae | *Gasteruption corniculigerum* | 14 | parasitoid |  |
| Bombyliidae | *Anthrax* sp.1 | 127 | parasitoid | √ |
| Bombyliidae | Bombyliidae sp.1 | 6 | parasitoid | √ |
| Bombyliidae | Bombyliidae sp.2 | 1 | parasitoid |  |
| Bombyliidae | Bombyliidae sp.3 | 1 | parasitoid | √ |
| Phoridae | Phoridae sp.1 | 7 | parasitoid | √ |
| Phoridae | Phoridae sp.2 | 1 | parasitoid | √ |
| Sarcophagidae | *Amobia auriceps* | 855 | parasitoid | √ |
| Sarcophagidae | *Amobia* sp.1 | 125 | parasitoid | √ |
| Sarcophagidae | *Miltogramma* sp.1 | 4 | parasitoid | √ |
| Sarcophagidae | *Miltogramma* sp.2 | 1 | parasitoid | √ |
| Sarcophagidae | *Miltogramma* sp.3 | 1 | parasitoid |  |
| Sarcophagidae | *Senotainia* sp.1 | 26 | parasitoid | √ |

Supplementary file 1b. Environmental correlates of host species community dissimilarity (NMDS on Morisita-Horn dissimilarity) across the study plots. Correlation coefficients, explained variation (r²) and probabilities (based on 1000 permutations) for the relationships between environmental variables and the NMDS axis scores. Significant P-values are indicated in bold.

| Vectors | Host species composition | | | |
| --- | --- | --- | --- | --- |
|  | NMDS1 | NMDS2 | r^2^ | Pr(>r) |
| Tree phylogeny NMDS1 | -0.9674 | 0.25327 | 0.0359 | 0.225 |
| Tree phylogeny NMDS2 | 0.26373 | -0.9646 | 0.1517 | **0.003** |
| Tree composition NMDS1 | 0.62106 | -0.78377 | 0.3298 | **0.001** |
| Tree composition NMDS2 | 0.16588 | 0.98615 | 0.0127 | 0.604 |
| Canopy cover | -0.99998 | -0.00593 | 0.5095 | **0.001** |
| Tree species richness | -0.90074 | -0.43436 | 0.0798 | 0.035 |
| Elevation | -0.65845 | 0.75262 | 0.13 | **0.005** |
| Eastness | 0.79555 | 0.60589 | 0.0586 | 0.079 |
| Northness | -0.07337 | -0.99731 | 0.0171 | 0.49 |
| Slope | -0.13253 | -0.99118 | 0.0836 | 0.031 |
| Tree FD (Rao's Q) | -0.97061 | 0.24067 | 0.0549 | 0.094 |
| Tree MPD | -0.93721 | -0.34878 | 0.1439 | **0.005** |
| Parasitoid phylogeny NMDS1 | 0.93994 | -0.34133 | 0.187 | **0.001** |
| Parasitoid phylogeny NMDS2 | -0.32919 | 0.94426 | 0.0183 | 0.462 |
| Parasitoid compositionNMDS1 | -0.94691 | 0.3215 | 0.1881 | **0.001** |
| Parasitoid compositionNMDS2 | 0.9524 | -0.30484 | 0.106 | **0.014** |
| stress=0.23 |  |  |  |  |

Supplementary file 1c. Environmental correlates of parasitoid species community dissimilarity (NMDS on Morisita-Horn dissimilarity) across the study plots. Correlation coefficients, explained variation (r²) and probabilities (based on 1000 permutations) for the relationships between environmental variables and the NMDS axis scores. Significant P-values are indicated in bold.

| Vectors | Parasitoid species composition | | | |
| --- | --- | --- | --- | --- |
|  | NMDS1 | NMDS2 | r^2^ | Pr(>r) |
| Tree phylogeny NMDS1 | 0.19179 | -0.98144 | 0.0217 | 0.422 |
| Tree phylogeny NMDS2 | -0.47649 | 0.87918 | 0.0518 | 0.12 |
| Tree composition NMDS1 | -0.55799 | 0.82985 | 0.2263 | **0.001** |
| Tree composition NMDS2 | -0.50111 | 0.86538 | 0.0201 | 0.418 |
| Canopy cover | 0.8741 | -0.48575 | 0.2736 | **0.001** |
| Tree species richness | 0.98384 | 0.17905 | 0.0524 | 0.122 |
| Elevation | 0.55299 | -0.83319 | 0.1263 | **0.007** |
| Eastness | -0.59888 | 0.80084 | 0.0717 | 0.045 |
| Northness | -0.81065 | 0.58554 | 0.0045 | 0.837 |
| Slope | 0.95482 | 0.29719 | 0.0176 | 0.507 |
| Tree FD (Rao's Q) | 0.88491 | -0.46576 | 0.0921 | **0.019** |
| Tree MPD | 0.99591 | 0.09035 | 0.0972 | **0.021** |
| Host phylogeny NMDS1 | -0.93007 | 0.36738 | 0.1688 | **0.016** |
| Host phylogeny NMDS2 | -0.93952 | -0.3425 | 0.0834 | **0.027** |
| Host compositionNMDS1 | -0.96573 | 0.25953 | 0.3706 | **0.001** |
| Host compositionNMDS2 | 0.52611 | -0.85042 | 0.0458 | 0.169 |
| stress=0.23 |  |  |  |  |

Supplementary file 1d. Environmental correlates of host phylogenetic community dissimilarity (NMDS on Morisita-Horn dissimilarity) across the study plots. Correlation coefficients, explained variation (r²) and probabilities (based on 1000 permutations) for the relationships between environmental variables and the NMDS axis scores. Significant P-values are indicated in bold.

| Vectors | Host phylogenetic composition | | | |
| --- | --- | --- | --- | --- |
|  | NMDS1 | NMDS2 | r^2^ | Pr(>r) |
| Tree phylogeny NMDS1 | 0.19179 | -0.98144 | 0.0217 | 0.386 |
| Tree phylogeny NMDS2 | -0.47649 | 0.87918 | 0.0518 | 0.128 |
| Tree composition NMDS1 | -0.55799 | 0.82985 | 0.2263 | **0.001** |
| Tree composition NMDS2 | -0.50111 | 0.86538 | 0.0201 | 0.433 |
| Canopy cover | 0.8741 | -0.48575 | 0.2736 | **0.001** |
| Tree species richness | 0.98384 | 0.17905 | 0.0524 | 0.100 |
| Elevation | 0.55299 | -0.83319 | 0.1263 | **0.001** |
| Eastness | -0.59888 | 0.80084 | 0.0717 | **0.04** |
| Northness | -0.81065 | 0.58554 | 0.0045 | 0.821 |
| Slope | 0.95482 | 0.29719 | 0.0176 | 0.507 |
| Tree FD (Rao's Q) | 0.88491 | -0.46576 | 0.0921 | **0.021** |
| Tree MPD | 0.99591 | 0.09035 | 0.0972 | **0.013** |
| Parasitoid phylogeny NMDS1 | -0.49168 | 0.87078 | 0.4286 | **0.001** |
| Parasitoid phylogeny NMDS2 | 0.61112 | -0.79154 | 0.0714 | 0.058 |
| Parasitoid compositionNMDS1 | 0.42314 | -0.90606 | 0.7623 | **0.001** |
| Parasitoid compositionNMDS2 | -0.95942 | -0.28198 | 0.2125 | **0.001** |
| stress=0.24 |  |  |  |  |

Supplementary file 1e. Environmental correlates of parasitoid phylogenetic community dissimilarity (NMDS on Morisita-Horn dissimilarity) across the study plots. Correlation coefficients, explained variation (r²) and probabilities (based on 1000 permutations) for the relationships between environmental variables and the NMDS axis scores. Significant P-values are indicated in bold.

| Vectors | Parasitoid phylogenetic composition | | | |
| --- | --- | --- | --- | --- |
|  | NMDS1 | NMDS2 | r^2^ | Pr(>r) |
| Tree phylogeny NMDS1 | -0.97914 | 0.20321 | 0.0305 | 0.274 |
| Tree phylogeny NMDS2 | 0.99965 | -0.02634 | 0.0889 | **0.024** |
| Tree composition NMDS1 | 0.82944 | -0.5586 | 0.2794 | **0.001** |
| Tree composition NMDS2 | 0.46615 | -0.8847 | 0.0789 | **0.031** |
| Canopy cover | -0.97111 | -0.23861 | 0.1497 | **0.004** |
| Tree species richness | -0.99834 | 0.05759 | 0.0594 | 0.094 |
| Elevation | -0.97095 | -0.23928 | 0.2242 | **0.001** |
| Eastness | 0.95102 | -0.30912 | 0.1682 | **0.001** |
| Northness | -0.27806 | -0.96056 | 0.0274 | 0.340 |
| Slope | -0.37915 | 0.92534 | 0.001 | 0.959 |
| Tree FD (Rao's Q) | -0.88976 | 0.45642 | 0.0819 | **0.031** |
| Tree MPD | -0.98331 | 0.18195 | 0.0363 | 0.223 |
| Host phylogeny NMDS1 | -0.2796 | -0.96012 | 0.0105 | 0.584 |
| Host phylogeny NMDS2 | -0.37192 | 0.92826 | 0.0022 | 0.914 |
| Host compositionNMDS1 | 0.92516 | 0.37957 | 0.1225 | **0.008** |
| Host compositionNMDS2 | -0.9934 | 0.11469 | 0.0485 | 0.138 |
| stress=0.20 |  |  |  |  |

|  | DF | Sum of Sqs | R^2^ | F | P |
| --- | --- | --- | --- | --- | --- |
| Canopy cover | 1 | 1.88 | 0.15 | 15.88 | 0.001 |
| Tree species richness | 1 | 0.18 | 0.01 | 1.53 | 0.131 |
| Elevation | 1 | 0.37 | 0.03 | 3.09 | 0.020 |
| Eastness | 1 | 0.10 | 0.01 | 0.87 | 0.502 |
| Northness | 1 | 0.10 | 0.01 | 0.85 | 0.490 |
| Slope | 1 | 0.32 | 0.03 | 2.74 | 0.016 |
| Tree FD (Rao’s Q) | 1 | 0.10 | 0.01 | 0.87 | 0.500 |
| Tree MPD | 1 | 0.10 | 0.01 | 0.85 | 0.493 |
| Residual | 76 | 9.00 | 0.74 |  |  |
| Total | 84 | 12.16 | 1.00 |  |  |

Supplementary file 1f. Results of permutational multivariate analysis of variance (PERMANOVA) between host species composition and environmental variables.

Supplementary file 1g. Results of permutational multivariate analysis of variance (PERMANOVA) between parasitoid species composition and environmental variables.

|  | DF | Sum of Sqs | R^2^ | F | P |
| --- | --- | --- | --- | --- | --- |
| Canopy cover | 1 | 1.14 | 0.08 | 7.72 | 0.001 |
| Tree species richness | 1 | 0.21 | 0.01 | 1.41 | 0.173 |
| Elevation | 1 | 0.40 | 0.03 | 2.71 | 0.015 |
| Eastness | 1 | 0.24 | 0.02 | 1.62 | 0.127 |
| Northness | 1 | 0.21 | 0.02 | 1.41 | 0.170 |
| Slope | 1 | 0.17 | 0.01 | 1.17 | 0.296 |
| Tree FD (Rao’s Q) | 1 | 0.19 | 0.01 | 1.30 | 0.252 |
| Tree MPD | 1 | 0.12 | 0.01 | 0.79 | 0.587 |
| Residual | 76 | 11.19 | 0.81 |  |  |
| Total | 84 | 13.87 | 1.00 |  |  |

Supplementary file 1h. Results of permutational multivariate analysis of variance (PERMANOVA) between host phylogenetic composition and environmental variables.

|  | DF | Sum of Sqs | R^2^ | F | P |
| --- | --- | --- | --- | --- | --- |
| Canopy cover | 1 | 0.00 | 0.01 | 0.90 | 0.385 |
| Tree species richness | 1 | 0.00 | 0.01 | 0.63 | 0.467 |
| Elevation | 1 | 0.00 | 0.00 | 0.31 | 0.666 |
| Eastness | 1 | 0.01 | 0.01 | 1.22 | 0.280 |
| Northness | 1 | 0.00 | 0.00 | 0.22 | 0.768 |
| Slope | 1 | 0.03 | 0.07 | 5.83 | 0.019 |
| Tree FD (Rao’s Q) | 1 | 0.00 | 0.01 | 0.53 | 0.507 |
| Tree MPD | 1 | 0.00 | 0.00 | 0.17 | 0.822 |
| Residual | 75 | 0.37 | 0.88 |  |  |
| Total | 83 | 0.42 | 1.00 |  |  |

Supplementary file 1i. Results of permutational multivariate analysis of variance (PERMANOVA) between parasitoid phylogenetic composition and environmental variables.

|  | DF | Sum of Sqs | R^2^ | F | P |
| --- | --- | --- | --- | --- | --- |
| Canopy cover | 1 | 0.01 | 0.04 | 3.91 | 0.013 |
| Tree species richness | 1 | 0.00 | 0.01 | 0.97 | 0.359 |
| Elevation | 1 | 0.00 | 0.02 | 1.96 | 0.116 |
| Eastness | 1 | 0.00 | 0.02 | 2.18 | 0.096 |
| Northness | 1 | 0.00 | 0.01 | 0.76 | 0.483 |
| Slope | 1 | 0.00 | 0.02 | 1.99 | 0.116 |
| Tree FD (Rao’s Q) | 1 | 0.00 | 0.01 | 0.86 | 0.393 |
| Tree MPD | 1 | 0.00 | 0.00 | 0.42 | 0.732 |
| Residual | 75 | 0.12 | 0.85 |  |  |
| Total | 83 | 0.14 | 1.00 |  |  |

Supplementary file 1j. Summary results of Mantel test between community composition of trees, hosts and parasitoids.

|  |  | Species composition |  |
| --- | --- | --- | --- |
|  | Tree & Host | Tree & Parasitoid | Host & Parasitoid |
| Mantel r | 0.13 | 0.14 | 0.65 |
| p | 0.001 | 0.001 | 0.001 |
|  |  |  |  |
|  |  | Phylogenetic composition | |
|  | Tree & Host | Tree & Parasitoid | Host & Parasitoid |
| Mantel r | 0.07 | 0.14 | 0.06 |
| p | 0.07 | 0.002 | 0.139 |

Supplementary file 1k. Summary results of alternative linear models for parasitoid generality, host vulnerability, robustness, linkage density, connectance, and interaction evenness of community-level host-parasitoid network indices across values of tree phylogenetic diversity (MPD). Standardized parameter estimates (with standard errors, t and P values) are shown for the variables retained in the minimal models.

|  |  | Est. | SE | t | P |
| --- | --- | --- | --- | --- | --- |
| Parasitoid generality | Intercept | 0.176 | 0.016 | 10.96 | <0.001 |
|  | Canopy cover | 0.033 | 0.016 | 2.03 | 0.046 |
| Host vulnerability | Intercept | 2.878 | 0.08 | 35.79 | <0.001 |
|  | Tree species richness: Site A | 0.072 | 0.13 | 0.57 | 0.569 |
|  | Tree species richness: Site B | 0.256 | 0.11 | 2.43 | 0.017 |
|  | Intercept | 0.631 | 0.01 | 83.44 | <0.001 |
| Robustness of parasitoids | Tree species richness: Site A | -0.023 | 0.01 | -1.95 | 0.055 |
|  | Tree species richness: Site B | -0.013 | 0.01 | -1.34 | 0.184 |
|  | Intercept | 2.039 | 0.04 | 50.98 | <0.001 |
| Linkage density | Elevation | -0.066 | 0.04 | -1.62 | 0.110 |
|  | Tree species richness: Site A | 0.091 | 0.06 | 1.42 | 0.160 |
|  | Tree species richness: Site B | 0.128 | 0.05 | 2.44 | 0.017 |
|  | Intercept | 0.511 | 0.009 | 59.12 | 0.025 |
|  | Canopy cover | -0.037 | 0.007 | -5.06 | <0.001 |
| Interaction evenness | Eastness | -0.018 | 0.007 | -2.50 | 0.015 |
